# Zerumbone Alleviates Neuropathic Pain through the Involvement of l-Arginine-Nitric Oxide-cGMP-K^+^ ATP Channel Pathways in Chronic Constriction Injury in Mice Model

**DOI:** 10.3390/molecules22040555

**Published:** 2017-03-30

**Authors:** Nurul Atiqah Zulazmi, Banulata Gopalsamy, Jasmine Chia Siew Min, Ahmad Akira Omar Farouk, Mohd Roslan Sulaiman, B. Hemabarathy Bharatham, Enoch Kumar Perimal

**Affiliations:** 1 Department of Biomedical Sciences, Faculty of Medicine and Health Sciences, University Putra Malaysia, 43400 Serdang, Selangor, Malaysia; atiqahzulazmi@yahoo.com (N.A.Z.); banulatagopalsamy@gmail.com (B.G.); enimsajsaj@gmail.com (J.C.S.M.); ahmadakira@upm.edu.my (A.A.O.F.); mrs@upm.edu (M.R.S.); 2 Department of Biomedical Sciences, School of Diagnostic and Applied Sciences, Faculty of Health Sciences, University Kebangsaan Malaysia, Jalan Raja Muda Abdul Aziz, 50300 Kuala Lumpur, Malaysia; hema@ukm.edu.my

**Keywords:** zerumbone, chronic constriction injury (CCI), neuropathic pain, nitric oxide, NO-cGMP pathway

## Abstract

The present study investigates the involvement of the l-arginine-Nitric Oxide-cGMP-K^+^ ATP pathways responsible for the action of anti-allodynic and antihyperalgesic activities of zerumbone in chronic constriction injury (CCI) induced neuropathic pain in mice. The role of l-arginine-NO-cGMP-K^+^ was assessed by the von Frey and the Randall-Selitto tests. Both allodynia and hyperalgesia assessments were carried out on the 14th day post CCI, 30 min after treatments were given for each respective pathway. Anti-allodynic and antihyperalgesic effects of zerumbone (10 mg/kg, i.p) were significantly reversed by the pre-treatment of l-arginine (10 mg/kg), 1H [1,2,4]Oxadiazole[4,3*a*]quinoxalin-1-one (ODQ), a soluble guanosyl cyclase blocker (2 mg/kg i.p.) and glibenclamide (ATP-sensitive potassium channel blocker) (10 mg/kg i.p.) (*p* < 0.05). Taken together, these results indicate that systemic administration of zerumbone produces significant anti-allodynic and antihyperalgesic activities in neuropathic pain in mice possibly due to involvement of the l-arginine-NO-cGMP-PKG-K^+^ ATP channel pathways in CCI model.

## 1. Introduction

Neuropathic pain has become a common issue that affects millions of people around the world [1]. Moreover, the Quick Reference Guide on Management of Cancer Pain, 2010, from the Ministry of Health in Malaysia, classified neuropathic pain as a challenging pain syndrome critically in need of adjuvant analgesics and additional interventions. The International Association Study of Pain (IASP) defines neuropathic pain as a pain that is caused by a lesion or disease on the somatosensory system. Thus, this type of pain is often associated with diseases or injuries to the peripheral and central nervous system that usually causes abnormal processing of sensory input [2,3].

Existing treatments such as non-steroidal anti-inflammatory drugs (NSAIDs) and opiates show little success with a limited response to neuropathic pain [4]. Additionally, commonly used medications for neuropathic pain result in numerous side effects, have unpredictable effectiveness, require complex dosing, with delayed analgesic onset, and also somehow reduces the patient’s quality of life [2,5].

Zerumbone is a bioactive sesquiterpene isolated from *Zingiber zerumbet* Smith. Zerumbone has been studied extensively in both in vivo and in vitro models [6]. Zerumbone has medicinal properties, with reports on its anti-nociceptive [7], anti-inflammatory [8] and, anti-tumor activities [9,10]. Our previous report showed that zerumbone possesses anti-allodynic and antihyperalgesic activities in chronic constriction injury (CCI) in animal models of neuropathic pain [11]. Other than that, we have recently reported the involvement of the serotonergic system in the anti-allodynic and antihyperalgesic activities of zerumbone in neuropathic pain [12]. Therefore, these findings suggest that the mechanism of action of zerumbone may also be linked to pathways associated with the neuropathic pain mechanism, one of which is the l-arginine-nitric oxide-cyclic guanosine monophosphate (cGMP)-K^+^ATP pathway.

l-arginine synthesizes nitric oxide (NO) from calcium-dependent constitutive isoforms of NO synthases (NOS)—neuronal NOS (nNOS), endothelial NOS (eNOS), or calcium-independent inducible NOS (iNOS) [13]. Previous reports have shown the involvement of NO in nociceptive synaptic transmission in both the central (CNS) and peripheral nervous systems (PNS) [13,14]. Increase in NO synthesis leads to release of excitatory neuropeptides, cytokines, and neurotransmitters. Indeed, NO is said to be involved in the maintenance of allodynia and hyperalgesia as NO synthases are up-regulated after nerve injury [14,15,16,17,18,19,20,21,22,23,24]. The enzyme guanylate cyclase (GC) is activated by NO, whereby its activation promotes the production of cGMP from GTP [25]. Previous studies have also suggested the possible role of the NO-cGMP pathway in activating other targets such as cGMP dependent protein kinase (PKG) and potassium channels (K^+^) [26,27,28,29]. Opening of potassium channels due to the l-arginine-NO-cGMP pathway allows regulation of the neuronal excitability through K^+^ ions permeating the membrane [30]. Studies have shown that opening of potassium channels results in antinociception [31,32].

On the basis of our previous work, zerumbone has shown to induce the antinociceptive effect in the chemical model of nociception in mice through the inhibition of the l-arginine-nitric oxide-cGMP-K^+^ATP channel pathways [7]. Hence, we hypothesized similar involvement of this pathway in the mechanism of action of zerumbone in neuropathic pain. This study attempts to verify the possible mechanisms of action of zerumbone through the involvement of NO/cGMP/K^+^ATP pathway linked to the anti-allodynic and antihyperalgesic activities of zerumbone, which could be a good prospect for the development of a new treatment in relieving neuropathic pain.

## 2. Results

### 2.1. l-Arginine-Nitric Oxide Pathway

The role of l-arginine-Nitric Oxide-Cyclic Guanosine Monophosphate-Potassium channel pathway in the anti-allodynic and antihyperalgesic effects of zerumbone were assessed by von Frey and Randall-Selitto tests. Both allodynic and hyperalgesic assessments were carried out on the 14th day post CCI, 30 min after their respective treatments were given.

**Figure 1:**
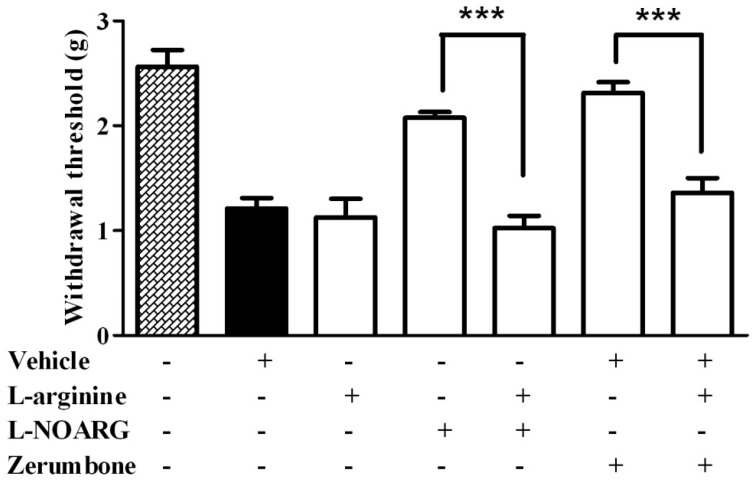
Effect of pre-treatment with l-arginine (10 mg/kg, i.p.) and l-NOARG (10 mg/kg, i.p.) on the anti-allodynic effect of zerumbone (10 mg/kg, i.p.) in CCI-induced neuropathic pain in mice. *** *p* < 0.001 comparing zerumbone to l-arginine + zerumbone treated groups and l-NOARG to l-arginine + l-NOARG treated groups. + or − indicate presence or absence of the respective treatment.

For the von Frey test, pre-treatment of l-arginine (10 mg/kg) 15 min before l-NOARG (10 mg/kg) showed significant effect in reversing anti-allodynic effect caused by l-NOARG alone (*p* < 0.001). Consequently, pre-treatment of l-arginine (10 mg/kg) intraperitoneally prior to zerumbone treatment significantly reversed the anti-allodynic effect of zerumbone (*p* < 0.001) (Figure 1).

**Figure 2:**
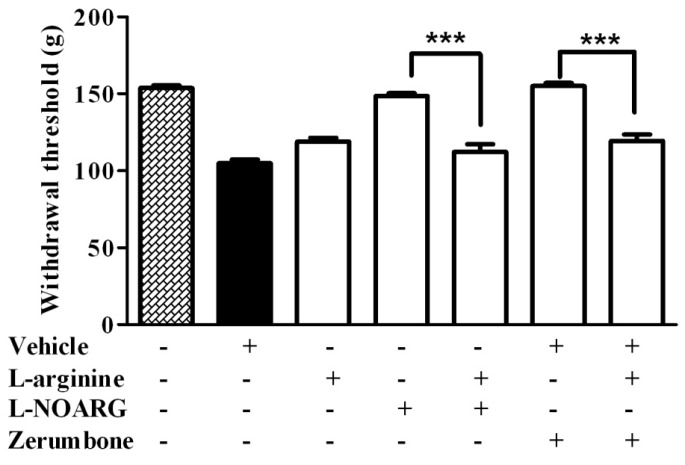
Effect of pre-treatment with l-arginine (10 mg/kg, i.p.) and l-NOARG (10 mg/kg, i.p.) on the antihyperalgesic effect of zerumbone (10 mg/kg, i.p.) in CCI-induced neuropathic pain in mice. *** *p* < 0.001 comparing zerumbone to l-arginine + zerumbone treated groups and l-NOARG to l-arginine + l-NOARG treated groups. + or − indicate presence or absence of the respective treatment.

Pre-treatment of l-arginine (10 mg/kg) with l-NOARG (10 mg/kg) for the Randall-Selitto test showed the similar effect to the von Frey test, as the hyperalgesic response was significantly increased (*p* < 0.001) when compared to the treatment of l-arginine alone. Similarly, pre-treatment of l-arginine (10 mg/kg) inhibited the antihyperalgesic effect of zerumbone (10 mg/kg) (*p* < 0.001) (Figure 2).

### 2.2. Cyclic Guanosine Monophosphate (cGMP) Pathway

**Figure 3:**
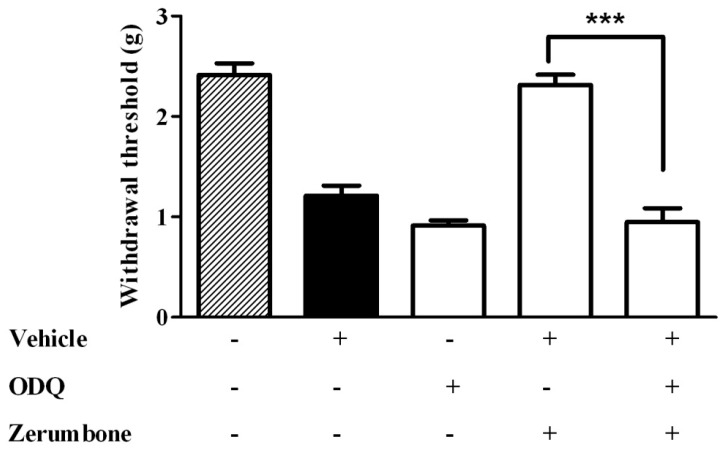
Effect of pre-treatment with ODQ (2 mg/kg, i.p.) on the anti-allodynic effect of zerumbone (10 mg/kg, i.p.) in CCI-induced neuropathic pain in mice. *** *p* < 0.001 comparing zerumbone to ODQ + zerumbone treated groups. + or − indicate presence or absence of the respective treatment.

**Figure 4:**
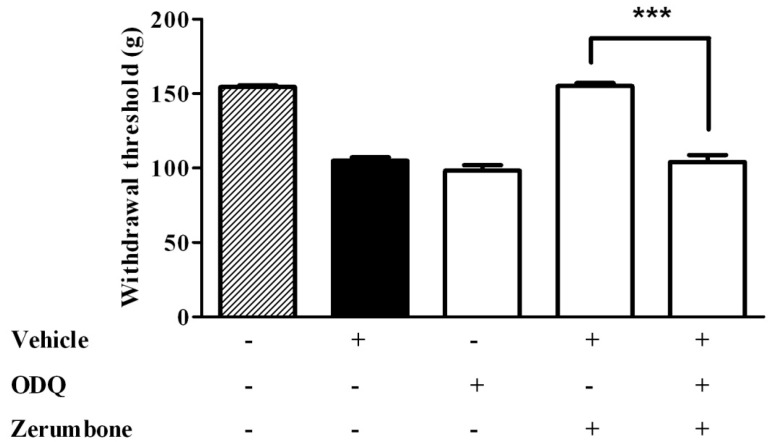
Effect of pre-treatment with ODQ (2 mg/kg, i.p.) on the antihyperalgesic effect of zerumbone (10 mg/kg, i.p.) in CCI-induced neuropathic pain in mice. *** *p* < 0.001 comparing zerumbone to ODQ + zerumbone treated groups. + or − indicate presence or absence of the respective treatment.

The cyclic GMP involvement was investigated by using the soluble guanosyl cyclase blocker (ODQ) and it demonstrated that cGMP is associated with the zerumbone anti-allodynic and antihyperalgesic effect. Pre-treatment of ODQ (2 mg/kg) with zerumbone significantly blocked the anti-allodynic effect of zerumbone (10 mg/kg) (*p* < 0.001) (Figure 3). Similarly, pre-treatment of ODQ (2 mg/kg) also reversed the antihyperalgesic effect caused by zerumbone (10 mg/kg) (*p* < 0.001) (Figure 4).

### 2.3. Potassium (K^+^) Channel Pathway

**Figure 5:**
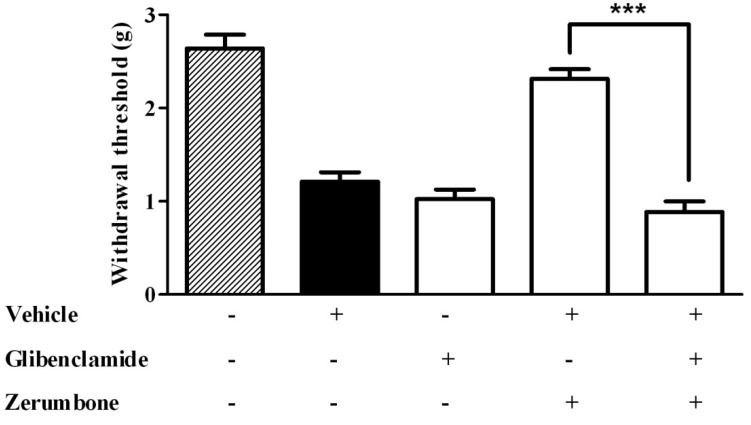
Effect of pre-treatment with glibenclamide (10 mg/kg, i.p.) on the anti-allodynic effect of zerumbone (10 mg/kg, i.p.) in CCI-induced neuropathic pain in mice. *** *p* < 0.001 comparing zerumbone to Glibenclamide + zerumbone treated groups. + or − indicate presence or absence of the respective treatment.

**Figure 6:**
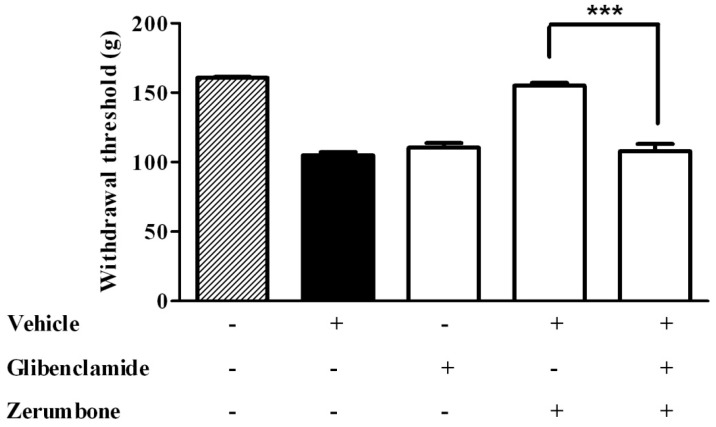
Effect of pre-treatment with glibenclamide (10 mg/kg, i.p.) on the antihyperalgesic effect of zerumbone (10 mg/kg, i.p.) in CCI-induced neuropathic pain in mice. *** *p* < 0.001 comparing zerumbone to Glibenclamide + zerumbone treated groups. + or − indicate presence or absence of the respective treatment.

In order to evaluate the role of the potassium K^+^ATP channel in effect of zerumbone, glibenclamide (10 mg/kg, i.p.), an ATP-sensitive K^+^ channel blocker, was injected 15 min prior to zerumbone. The data recorded clearly showed the involvement of the K^+^ATP channel in the anti-allodynic effect of zerumbone (10 mg/kg), where glibenclamide significantly reversed the effect of zerumbone (*p* < 0.001) (Figure 5). In the similar experimental design, pre-treatment of glibenclamide (10 mg/kg, i.p.) significantly reversed the antihyperalgesic effect of zerumbone (10 mg/kg, i.p.) (*p* < 0.001) (Figure 6).

### 2.4. Rota Rod Analysis

**Figure 7:**
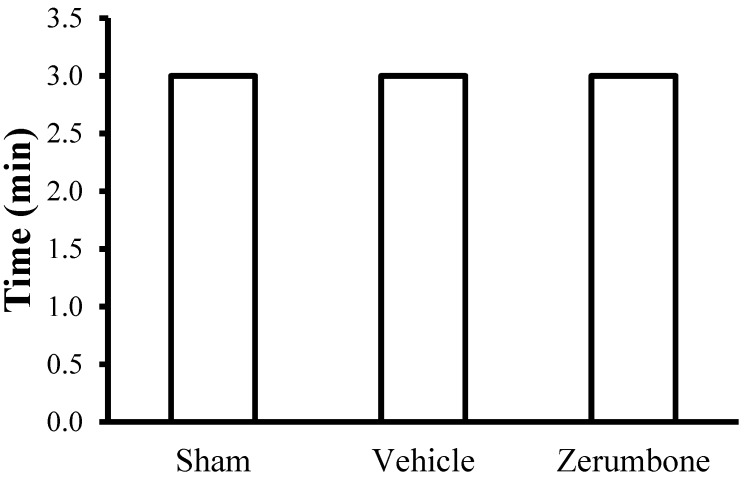
Rota rod analysis to test the possible sedative effect following the treatment of zerumbone (10 mg/kg; i.p).

In order to evaluate the sedative effect of treatments, mice from the sham, vehicle and zerumbone (10 mg/kg, i.p.) groups, were tested for their ability to balance on the rotating bar. The figure (Figure 7) clearly depicts that all the mice from the three groups were able to maintain their balance throughout the entire length of testing.

## 3. Discussion

The detail and exact mechanisms that underlies both anti-allodynic and antihyperalgesic effects of zerumbone has not yet been elucidated. However, it seems that different pathways may be involved in the antineuropathic activity of zerumbone. In our current study, we further characterize the mechanisms involved in the effects of zerumbone and demonstrated the involvement of NO-cGMP-K^+^ in the antihyperalgesic and anti-allodynic effects of zerumbone.

Even though NO modulators are known to play an important role in neuropathic conditions, the detailed mechanisms are still unclear. Nitric oxide synthesis has been reported to produce hyperalgesia after the activation of NMDA receptors [33]. Synthesis of NO is crucial in the maintenance of neuropathic pain. Research has shown that compression or inflammation at the nerve tissue causes an up-regulation of NOS and NO formation in the spinal cord [34]. In pathologic conditions, the three well-characterized isoforms of NO synthase—nNOS, eNOS, iNOS could also be up-regulated in nervous tissues [35,36,37]. The expression of nNOS in sensory neurons and iNOS in macrophages and Schwann cells are up-regulated following peripheral nerve injury [38,39].

l-arginine is a basic semi-essential amino acid [40] and is a precursor for the production of nitric oxide (NO). NO is produced from the oxidation of terminal guanidine nitrogen of l-arginine, which is then converted to l-citrulline by NOS in mammalian cells [41]. Previously, it has been reported that administration of l-arginine caused increased hyperalgesia and enhanced nociceptive or inflammatory responses evoked by bradykinin, substance P, and dextran [42,43]. Based on the current findings, the analgesic action of zerumbone was reversed when the animals were pre-treated with l-arginine, at a dose that did not produce any significant changes to the CCI-induced nociception, clearly indicating the involvement of nitric oxide in the antineuropathic effects of zerumbone.

We supported the experiment by using inhibitors of NOS, which cause a decrease in NO synthesis. N^G^-nitro-l-arginine (l-NOARG), is an active eNOS and nNOS inhibitor that has been widely used to attenuate constitutive NO [44]. The pharmacokinetics of l-NOARG showed that the drug has a mean residence time of about 30 h. The plasma concentration of l-NOARG declines in a biexponential trend with the average half-life of 11.0 ± 3.1 min (rapid declining phase) and 20.0 ± 4.9 h (slowly declining phase). l-NOARG has a volume distribution of 2.5 L/kg, causing it to be extensively distributed to extravascular tissues even though it insignificantly binds to the plasma protein. The prolonged duration of action of l-NOARG that is reported in some in vivo studies is due to the slow elimination. l-NOARG has minimal urinary excretion of unchanged l-NOARG whereby its excretion is mainly by the metabolism or biliary excretion [45].

At the dose of 10 mg/kg, l-NOARG produced significant anti-allodynic and antihyperalgesic effects on the CCI model, however, the pre-administration of l-arginine significantly reversed its anti-allodynic and antihyperalgesic activities. Administration of zerumbone produced a similar pattern as with l-NOARG. The results are supported by previous studies whereby a NOS inhibitor systemically reduces thermal hyperalgesia produced by glutamate [46], and relieves mechanical allodynia in CCI-induced neuropathic rats [47]. NOS inhibitors have also been shown to be involved in many other compounds investigated as analgesics for neuropathic pain [48,49]. This further implies the involvement of NO in mediating the anti-allodynic and antihyperalgesic effects of zerumbone by inhibiting the synthesis of NO.

The production of NO consequently stimulates an increase in the production of intracellular cGMP through soluble guanylate cyclase activation [50]. Soluble guanylate cyclase converts guanosine triphosphate to cGMP. The NO-cGMP pathway modulates intracellular processes through activation of protein kinases, phosphodiesterases, and ion channels, which results in alterations to the K^+^ and Ca^2+^ currents [51,52,53,54,55]. Our present study managed to demonstrate the involvement of the cGMP pathway through the reversal of the anti-allodynic and antihyperalgesic activities of zerumbone using ODQ, a soluble guanylate cylcase inhibitor. Similar effects have been reported with analgesics such as dipyrone and diclofenac [56,57].

Experimental data have indicated a link between the activation of the NO-cGMP pathway and the opening of the ATP-sensitive K^+^ channels[58]. From the results observed, the ATP-sensitive K^+^ channel is suggested to be involved in the mechanism of action of zerumbone. Pre-treatment with glibenclamide, an ATP-sensitive K^+^ channel blocker, significantly reversed both anti-allodynic and antihyperalgesic activities of zerumbone. Zerumbone possibly acts by modulating K^+^ currents through the efflux of K^+^ ions permeating the membrane. Increase in K^+^ ion efflux alters the membrane potential to avert from action potential generation, which results in the decrease of neurotransmitter release [30]. Other than that, the effect of zerumbone through the activation of the NO-dependent pathway is similar to some pharmacological studies that have evaluated NO/cGMP activation and the opening K^+^ channels, which relates to the opioidergic pathway [59].

These results are in agreement with the recent article reported by our group stating the possible involvement of nitric oxide-cGMP pathways in the antinociceptive effects of zerumbone [7,60]. This displays the potent inhibition of zerumbone towards NO through inhibition of NOS which enlightens us on the mechanism of action of zerumbone in neuropathic pain. In addition, previous research has reported that zerumbone managed to reduce the overexpression of inducible nitric oxide synthase (iNOS) and consecutively, reduce the production of NO [9,61]. Most data from previous studies has reported the importance of iNOS [62] in the pathogenesis of nerve injury-induced neuropathic pain. Local expression of iNOS starts as early as the third day and maintains expression for at least 26 days at the constriction sites after nerve injury [22,63,64,65]. Moreover, it slows down the regeneration of myelinated and smaller fibres at the site of the injury [63]. Therefore, zerumbone possibly exerts its effects by inhibition of iNOS, which might help in the nerve reparation process after nerve injury.

Nitric oxide can be classified as a free radical that can be dangerously combined with superoxide anions to form hydroxyl free radicals [66]. Several journals have reported that locally applied l-NAME may possibly reduce the levels of these toxic free radicals at the site of CCI, resulting in the reduction of thermal hyperalgesia [67]. Similarly, we speculate that zerumbone reduces these free radicals due to its dual anti-oxidant and anti-inflammatory properties [68]. Indeed, zerumbone was able to suppress free radicals (superoxide anion) generation from NADPH oxidase xanthine oxidase, expression of iNOS (inducible nitric oxide synthase) and COX (cyclooxygenase)-2, as well as the release of TNF-α [9].

Zerumbone was administered intraperitoneally and the presence of this compound is able to systemically alleviate the symptoms of allodynia and hyperalgesia. However, the exact site of action is relatively unclear, due to the lack of reports on the pharmacological properties of zerumbone. Speculation could be made that zerumbone might act by reducing inflammation, lowering the sensitivity at nerve terminals, or might be directly involved in the inhibition of signal transduction at the different levels of the pain pathway which includes peripheral nociceptors, dorsal root ganglion on the spinal cord. This represents a new approach in discovering new therapeutics for neuropathic pain [69,70]. Therefore, taking previous findings and our current data into account, we suggest that the analgesic effect of zerumbone is partly due to the activation of the l-arginine/NO/cGMP/ATP-sensitive K^+^ channel pathway in the CCI-induced neuropathic pain in animal model.

## 4. Materials and Methods

### 4.1. Experimental Animals

Male ICR mice (25–35 g) were used in this study. All mice (*n* = 8 mice in each group) were housed under 12 h light-dark cycles with the environment maintained at 20–24°C. The animals were allowed free access to tap water and commercial pellet. All experiments were conducted between 8:00 a.m. and 5:00 p.m. The handling of mice was based on Zimmermann [71] in accordance with Ethical Guidelines for Investigation of Experimental Pain in Conscious Animals as issued by the International Association for the Study of Pain. The protocol and procedure of the experiment has been approved by Institutional Animal Care and Use Committee (IACUC) UPM (UPM/IACUC/AUP-R060/2013).

### 4.2. Surgical Procedure

Neuropathic pain was induced in mice by performing chronic constriction injury (CCI) of the common sciatic nerve as previously described by Bennett and Xie [72,73]. The CCI model has proven to be the best animal model, compared to other models, as it produces the most sustained response in peripheral nerve injury [4]. Briefly, mice were deeply anesthetized with tribromoethanol (250 mg/kg, i.p.) and the fur on the left thigh region was shaved. The left common sciatic nerve was isolated from adherent tissue and exposed at the mid-thigh by blunt dissection through the biceps femoris.

The common sciatic nerve that is proximal to the trifurcation was loosely ligated with one ligature using chromic silk suture Deme Tech until a slight twitch was observed in the expected hind limb. Then, the skin layer was immediately sutured with the absorbable synthetic braided suture Vigilenz (Brilon, Germany). Lastly, iodine was applied externally with a cotton swab on the site of the incision. Another group that consisted of sham mice were used for lesion-injured mice or the control group. This group of animals were subjected to the same procedure, excluding the ligation of the sciatic nerve.

### 4.3. Preparation of Zerumbone for Experiments

Zerumbone was prepared by dissolving in dimethylsulfoxide (DMSO), Tween 20 and normal saline (0.99% NaCl) in a 5:5:90 (*v*/*v*/*v*) fraction. The final concentration of DMSO did not exceed 5% and caused no detectable effect by itself.

### 4.4. Preparation of Drugs and Chemicals

Nω-nitro-l-arginine (l-NOARG), l-arginine hydrochloride (l-arginine), glibenclamide, DMSO, and 1-*H*-[1,2,4]Oxadiazole[4,3-a] quinoxaline-1-one (ODQ) were purchased from Sigma-Aldrich Chemical Co. (St. Louis, MO, USA). All chemicals were dissolved in saline solution (0.99% NaCl). All drugs were freshly prepared before experiments and administered intraperitoneally in a volume of 10 mL/kg unless stated otherwise.

### 4.5. Groups and Timeline

**Figure 8:**
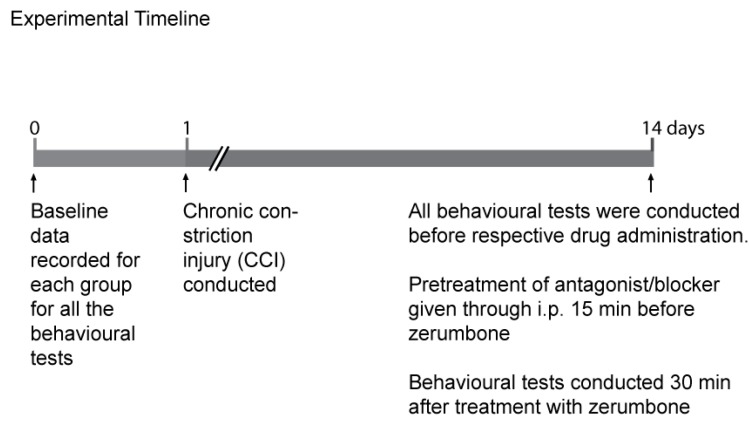
Timeline for the involvement of the l-arginine-Nitric Oxide-cGMP-K^+^ATP pathway in zerumbone anti-allodynic and antihyperalgesic activities.

A total of 96 mice with 8 mice per group was used. The response for tactile allodynia and mechanical hyperalgesia for every group (Table 1) was recorded for every mouse before CCI (baseline) and on post-surgery day 14 (Figure 8). On the 14th day the pre-treatment substances were administered according to its respective groups which were l-arginine (10 mg/kg, i.p.), l-NOARG (10 mg/kg, i.p.), ODQ (2 mg/kg, i.p.), and Glibenclamide (10 mg/kg, i.p.). The administration of the drugs by injection was 15 min prior to the administration of 10 mg/kg of zerumbone. Data for both of the behavioural tests were recorded 30 min after the administration of the final treatment.

### 4.6. Allodynia Effect

Tactile allodynia was evaluated by measuring the hind paw withdrawal response to a semi-flexible von Frey filament using an Electronic von Frey Anesthesiometer (IITC, Woodland Hills, CA, USA). Mice were placed individually in clear Plexiglas boxes on top of a wire mesh grid to allow the access to the ventral surface of the hind paws. Prior to the test, mice were allowed to habituate for at least 10 min, until their exploratory behaviour diminished before stimulation was initiated. A semi-flexible probe was applied vertically to the midplantar left and right hind paws with a gradual increase in pressure until the paw was withdrawn or elevated slowly which indicated a maximal force. Hence, the maximum applied pressure was noted when the probe was retracted. Both ipsilateral and contralateral hind paws were tested. These steps were repeated three times and the average measurement was calculated and recorded [74]. Mice that exhibited motor deficits during the pre-operative day and the post-operative days were excluded from further study.

### 4.7. Hyperalgesia Effect

The response towards mechanical hyperalgesia was assessed according to a Randall-Selitto Pressure Analgesiometer (IITC, Woodland Hills, CA, USA) based on the method previously described by [75]. The mice were habituated to the testing procedure a day before the experiment. They were restrained using a clean cloth and one paw at a time was placed in between the pressure applicator. An incremental pressure was applied gently onto the dorsal surface of the ipsilateral and contralateral paw, and the pressure (g) that showed the first nociceptive response such as squealing or paw withdrawal was recorded as the pain threshold. The stimulus was applied on both paws with a cut-off pressure of 250 g. Mice that exhibited motor deficits during the pre-operative day or the post-operative days were excluded from further study.

### 4.8. Rota Rod Analysis

Rota rod analysis was carried out to test the possible sedative effect of zerumbone treatment. Mice in the sham, vehicle, and zerumbone groups were placed on the rota rod (UgoBasile, Varese, Italy) bar rotating at 20 rpm, 30 min after drug administration. The time spent by the mice on the rotating bar was recorded and a cut-off time of 3 min was employed after which the mice were returned to their respective home cages.

### 4.9. Statistical Analysis

Results are reported as mean ± standard error (S.E.) for each group. Parametric values were analysed using one way analysis of variance (ANOVA) followed by Tukey’s *pos hoc* test using GraphPad-Prism v5.0 software (GraphPad, San Diego, CA, USA). The significance difference is indicated as * *p* < 0.05, ** *p* < 0.01, *** *p* < 0.001.

## 5. Conclusions

Zerumbone displayed inhibition to nitric oxide production, which can indirectly reduce the activation of NOS. The presence of cGMP is essential for zerumbone to exhibit its anti-allodynic and antihyperalgesic activities. Zerumbone is also suggested to exert its activity by the opening of K^+^ channels that help in reducing membrane excitability. In brief, we conclude that the anti-allodynic and antihyperalgesic activity of zerumbone is mediated through the l-arginine/NO-cGMP/ATP-sensitive K^+^ channel pathway. These findings suggest that zerumbone acts as a promising novel therapeutic agent for neuropathic pain. More extensive studies are needed to elucidate the detailed mechanism of action involved in zerumbone.

## Figures and Tables

**Table 1:** Experimental Groups.

Experimental Groups (*n* = 6)	Dose	Experimental Conditions
Sham	-	Without ligature to the nerve and no treatment
Vehicle (mL/kg, i.p.)	10	Subjected to CCI and treated with vehicle
Zerumbone (mg/kg, i.p.)	10	Subjected to CCI and treated with zerumbone
l-arginine (mg/kg, i.p.)	10	Subjected to CCI and pre-treated with different antagonist and agonist
l-NOARG (mg/kg, i.p.)	10	
l-arginine + l-NOARG	10 + 10	
l-arginine + Zerumbone	10 + 10	
l-NOARG + Zerumbone	10 + 10	
ODQ (mg/kg, i.p.)	2	Subjected to CCI and pre-treated with ODQ
ODQ + Zerumbone	2 + 10	
Glibenclamide (mg/kg, i.p.)	10	Subjected to CCI and pre-treated with Glibenclamide
Glibenclamide + Zerumbone	10 + 10	
